# A Possible Case of Cherubism in a 17^th^-Century Korean Mummy

**DOI:** 10.1371/journal.pone.0102441

**Published:** 2014-08-05

**Authors:** Israel Hershkovitz, Mark Spigelman, Rachel Sarig, Do-Sun Lim, In Sun Lee, Chang Seok Oh, Hila May, Elisabetta Boaretto, Yi-Suk Kim, Soong Deok Lee, Nathan Peled, Myeung Ju Kim, Talya Toledano, Gila Kahila Bar-Gal, Dong Hoon Shin

**Affiliations:** 1 Department of Anatomy and Anthropology, Sackler Faculty of Medicine, Tel-Aviv University, Tel-Aviv, Israel; 2 Department of Dental Hygiene, Eulji University, Seongnam, Gyeonggi-do, Korea; 3 Department of Diagnostic Radiology, Seoul National University College of Medicine, Seoul, Korea; 4 Department of Anatomy, Seoul National University College of Medicine, Seoul, Korea; 5 Institute of Forensic Medicine, Seoul National University College of Medicine, Seoul, Korea; 6 Department of Forensic Science, Seoul National University College of Medicine, Seoul, Korea; 7 D-REAMS Radiocarbon Dating Laboratory, Weizmann Institute of Science, Rehovot, Israel; 8 Department of Anatomy, Ewha Womans University School of Medicine, Seoul, Korea; 9 Department of Radiology, Carmel Medical Center, Haifa, Israel; 10 Department of Anatomy, Dankook University College of Medicine, Yongin-si, Gyeonggi-do, Korea; 11 Department of Radiology, Maimonides Medical Center, Brooklyn, New York, United States of America; 12 Department of Virology, Koret School of Veterinary Medicine, Hebrew University of Jerusalem, Rehovot, Israel; University of Delaware, United States of America

## Abstract

Cherubism is a benign fibro-osseous disease of childhood limited specifically to the maxilla and mandible. The progressive replacement of the jaw bones with expansile multilocular cystic lesions causes eventual prominence of the lower face, and hence the classic “cherubic” phenotype reflecting variable extents of jaw hypertrophy. Histologically, this condition has been characterized as replacement of the normal bone matrix with multicystic pockets of fibrous stroma and osteoclastic giant cells. Because of radiographic features common to both, primarily the presence of multiloculated lucencies with heterogeneous “ground-glass” sclerosis on CT imaging, cherubism was long mistaken for a craniofacial subtype of fibrous dysplasia. In 1999, however, the distinct genetic basis for cherubism was mapped to chromosome 4p16.3 and the SH-3 binding protein SH3BP2. But while there are already three suspected cases of fibrous dysplasia amongst archaeological populations, no definitive cases of cherubism have yet been reported in historical populations. In the current study we describe micro- and macro-structural changes in the face of a 17^th^ century Joseon Dynasty Korean mummy which may coincide with the clinic-pathologic and radiologic features of cherubism.

## Introduction

First described by Jones in 1933 as “familial multilocular cystic disease of the jaws,” cherubism is a benign fibro-osseous disease of childhood limited specifically to the maxilla and mandible without involvement of the non-facial bones [1]. The more familiar term “cherubism” was coined by Jones in reference to the classic phenotype of symmetrically rounded cheeks and upturned-eyes of affected individuals, features which are reminiscent of the cherubs depicted in Renaissance art. The clinical progression of the disease typically begins with a painless expansion of the jaw in a child aged 2–5 years, and continues to adolescence with variable levels of jaw hypertrophy. The lesions begin to spontaneously stabilize and regress at puberty, and eventually sclerose in adulthood. By the mid-20's, residual deformity of the jaws is rarely seen, if at all [2]–[7].

The histological basis of cherubism was not initially addressed by Jones, but has since been characterized as replacement of the bone matrix with multilocular cysts composed of proliferating fibrous stromal cells and multinucleated osteoclastic giant cells. The most widely accepted proposal for the pathogenesis of this condition involves perivascular fibrosis inducing mesenchymal abnormalities and decreased oxygenation [3], [8].

The anatomic distribution of cherubism is defined by its primary involvement of the mandible, accompanied in most cases by maxillary lesions of variable severity. The disease usually begins near the angle of the mandible, expanding into the body and ramus, and often sparing the condyles. Mandibular involvement is typically bilateral, and preservation of the mandibular condyles is considered pathognomonic of cherubism. Progressive replacement of the jaw bones with expansile multilocular cystic lesions causes eventual prominence of the lower face, and hence the “cherubic” phenotype reflecting jaw hypertrophy [8]. The phenotype ranges from absence of clinical manifestations to forms complicated by severe mandibular and maxillary overgrowth with respiratory, vision, speech, and swallowing problems. A severe level of jaw hypertrophy can ultimately affect the dentition, leading to missing, malformed, and/or abnormally spaced teeth [9]. Bones other than the jaws are usually not affected and the individual is otherwise normal.

The radiologic hallmarks of cherubism involve replacement of the bony matrix with symmetrical multiloculated radiolucencies with a ground-glass appearance of variable sclerosis on CT imaging. The lesions will typically manifest as cysts with scalloped borders, filled with soft-tissue density material, causing bony expansion, most prominently of the outer cortex [9].

Cherubism was initially characterized as familial, albeit sporadic cases have since been described. Because of radiographic features common to both, the condition has been specifically described as a hereditary craniofacial subtype of fibrous dysplasia. In 1999, however, a distinct genetic basis for cherubism was established, thereby cleaving it from its erroneous title of “fibrous dysplasia of the jaw.” Following the mapping of cherubism to chromosome 4p16.3 [10]–[14], Ueki et al. [13] traced the development of the disorder to a series of point mutations causing amino acid substitutions in the SH-3 binding protein SH3BP2 [12]. Since then, mutations in the *SH3BP2* gene have been identified in approximately 80 percent of people suffering from cherubism [13], [14]. Despite the mapping of a specific causative gene, a propensity toward cherubism in an individual with no family history of this disorder would suggest that a *de novo* genetic mutation is involved. The proportion of cases caused by such mutations is unknown, since cherubism displays variable expressivity and incomplete penetrance [12].

No definitive cases of cherubism have yet been reported in historical populations. Three suspected cases of fibrous dysplasia from archaeological populations have been reported. The first case suspected with this condition was of a male skeleton from a pre-Colombian site in Illinois. In this case the disease affected the bones on the left side of the body including skull femur, tibia, fibula, foot bones, pelvis, ribs and vertebrae. The left side of the skull was deformed and the long bones manifested thin cortex, multiple cyst-like lesions and poorly organized bone. His foot bones, pelvis, ribs and 6^th^ cervical vertebra demonstrated increased porosity of the cortical surface [15].

Wells [16] described a case of polyostotic fibrous dysplasia in a 7^th^ century male from an Anglo-Saxon site in Britain. His left humerus was strongly bowed laterally with irregular expansions throughout the shaft. The radiological examination lent support to this diagnosis revealing an enlarged cortex, abnormal growth of the spongy bone with cystic cavities between the woven bone and the cortex. Further archeological evidence for fibrous dysplasia was found in an Egyptian female skull from a 12^th^ Dynasty site near Matanieh in Upper Egypt. In this case, dense round nodules were found near the frontal sinus with coarse bony spicules covering the internal surface of the sinus [17].

The aims of the current study are to report on morpho-structural alternations in the lower face of a 17^th^ century Korean mummy, to provide a range of diverse information (aDNA, macro- and micro-structural features) that may be compatible with the clinicopathologic and radiologic features of cherubism, and thus establish the first case of the disease in a historic population.

## Materials and Methods

**Figure 1:**
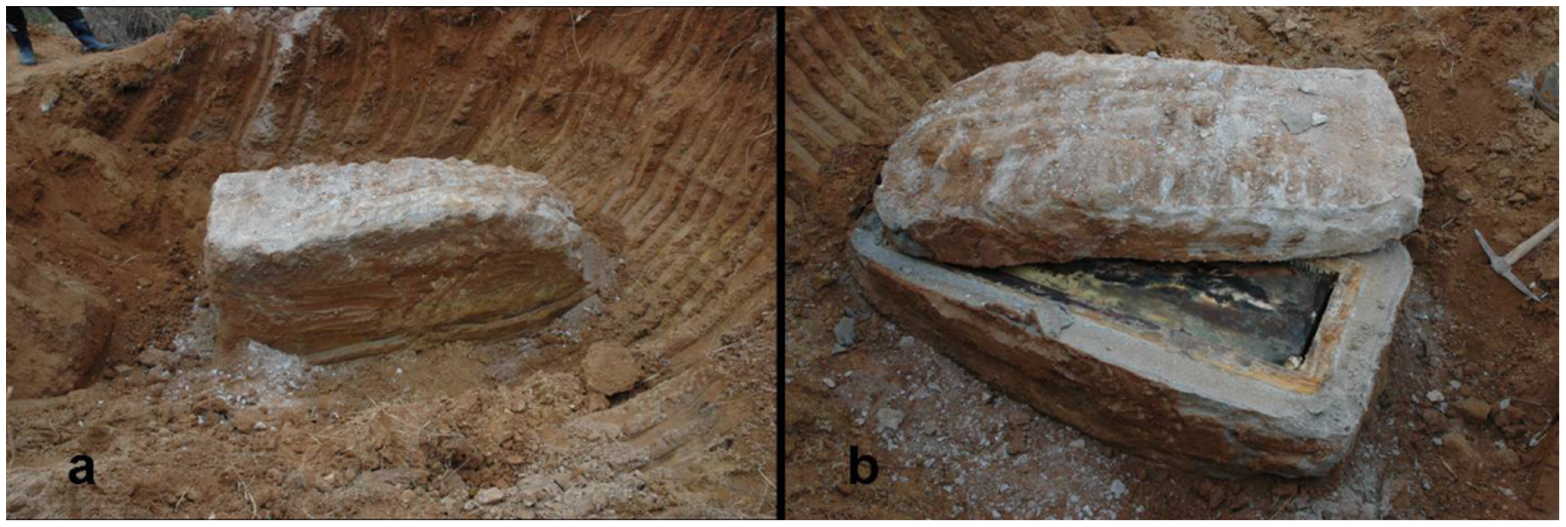
A lime-soil mixture barrier (LSMB) tomb of the Joseon Dynasty period, Korea. The size of the whole block is 2.3(length) ×1.0 m (width) ×1.0 m (height) (A). A cut through the lime capsule revealing the inner coffins (B).

**Figure 2:**
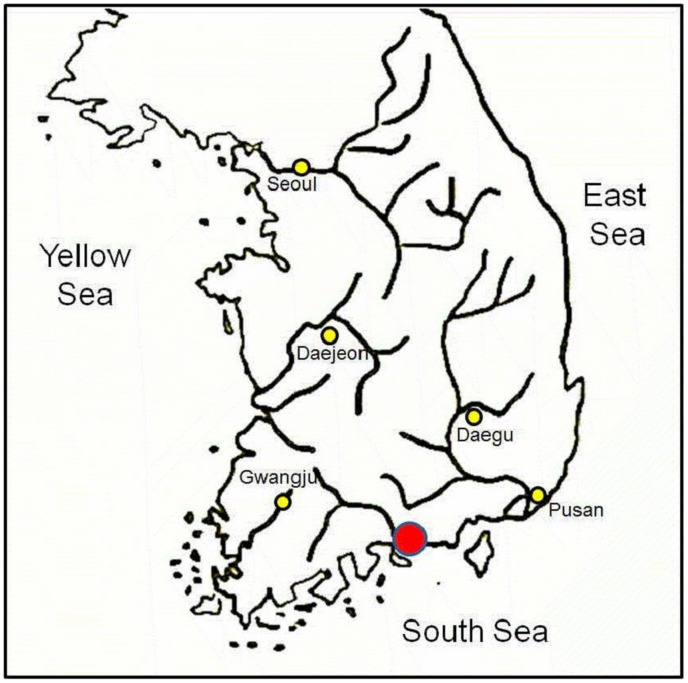
The location where the mummy (Hadong I) was found: Hadong, a coastal county where the Seomjin River flows into the Korean South Sea (red circle). Yellow dots indicate the modern major cities of Korea.

**Figure 3:**
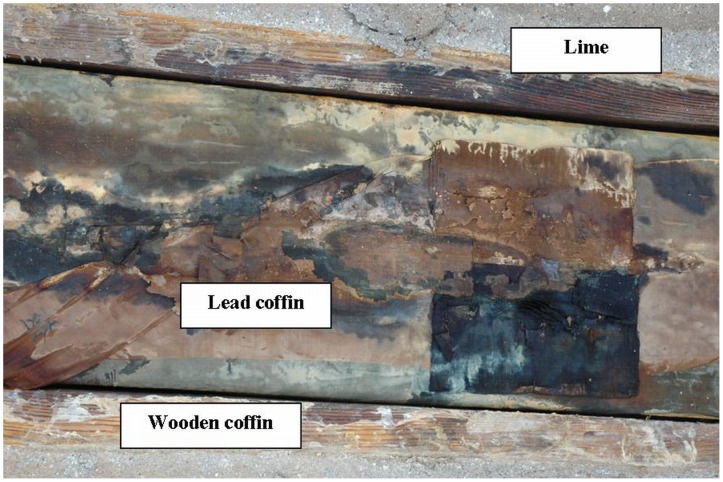
The coffin of the mummy. Note that the coffin was encapsulated by lime and was of the double type, namely an external wooden coffin and internal lead coffin.

**Figure 4:**
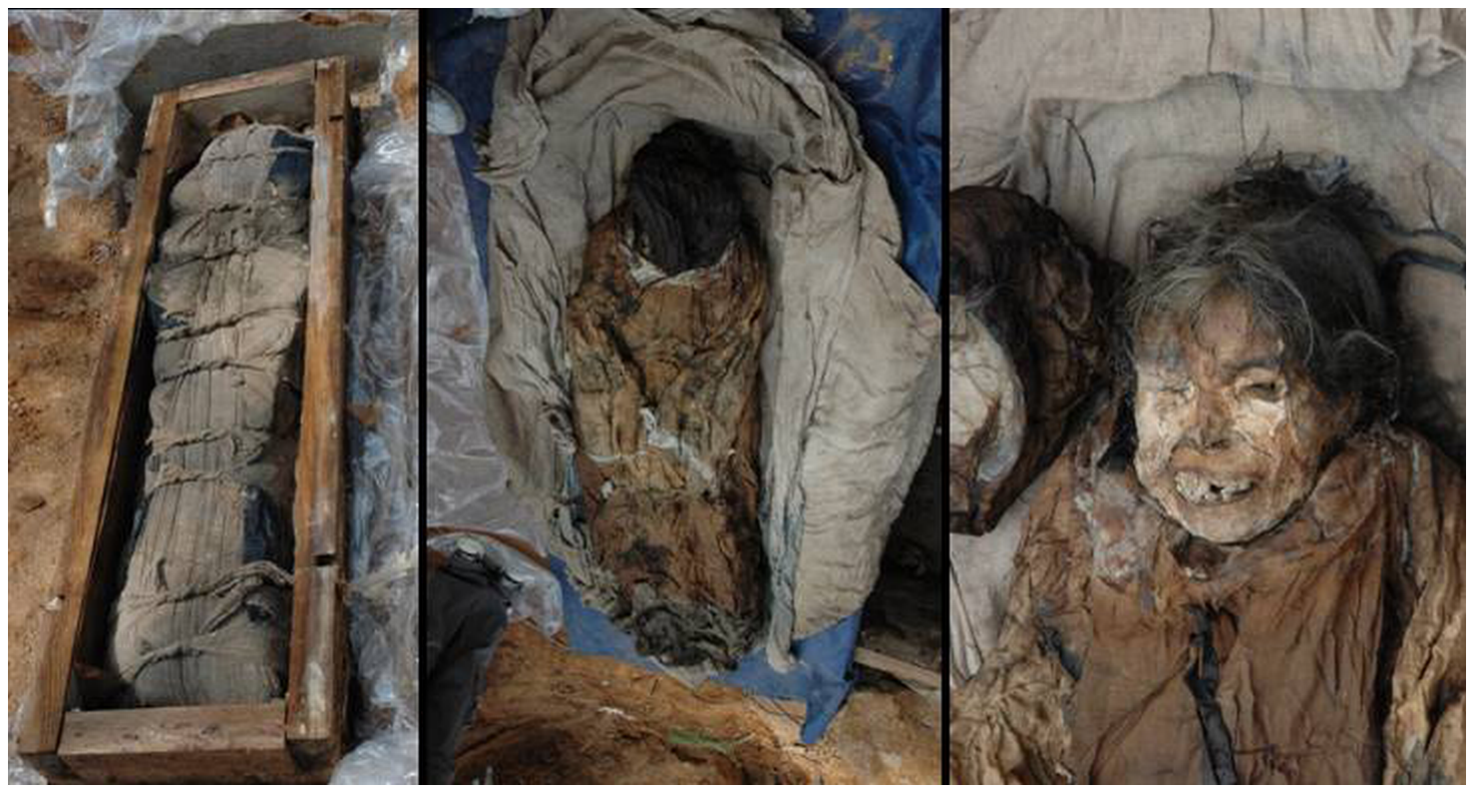
A clothes-wrapped body found inside the coffin: Left-the wrapped body inside the coffin; Center-the body still with its clothes, note that the face is covered by a veil; Right-the mummified body, close-up of the face.

On 6 April 2006, a female mummy was found within a lime-soil mixture barrier (LSMB) tomb (Fig. 1). The isolated tomb was excavated at the construction site of a thermoelectric power plant in Hadong, a coastal county where the Seomjin River flows into the Korean South Sea (Fig. 2). Historians and archaeologists alike have attributed the tomb to the 17th century AD, a Joseon Dynasty tomb probably belonging to the "Sungju Lee" clan: LSMB tombs were officially endorsed as a burial system for the ruling Korean elite during this dynasty [18]. The appearance of this type of burial practice is associated with the rise of Neo-Confucians as a political power in Korea from the 14th century onward [19]. Until the Joseon Dynasty, Buddhists dominated Korea politically, economically and culturally. In their efforts to topple the Buddhists' hegemony, the Neo-Confucians promoted reforms in almost every aspect of Korean life, including funerary and burial practices. The coffin found in Hadong was encapsulated by hard cemented lime and was of the “double type”, namely an external wooden coffin and internally a lead coffin (Fig. 3). This burial procedure, especially the cemented hard rock capsule around the coffin, was intended to deter intruders. Opening the lid of the inner coffin exposed a clothes-wrapped body (Fig. 4). Upon lifting the mummy out of the inner coffin, various cultural artifacts were found at the bottom of the inner coffin, including a funeral banner, wood and clay figurines, a mask, pillow and a silk tabby [18]. All the finds together with the burial shroud were moved to the Andong National University Museum (ANUM) for further analysis. The mummy was transferred to the Laboratory of Bio-anthropology and Paleopathology, Institute of Forensic Medicine, Seoul National University College of Medicine for anthropological study. Finally, although many mummies from the Joseon Dynasty period have been found in Korea, only 18 have been thoroughly studied [20].

All necessary permits were obtained for the subsequent study, which complied with all current relevant regulations. The study was performed in accordance with “The Vermillion Accord on Human Remains, World Archaeological Congress, South Dakota, 1989." Consent for the study of this individual was sought from and given by her descendants during the archaeological investigation. In addition, the study was approved by the Institutional Review Board (IRB) of the Seoul National University College of Medicine and certified to the Laboratory of Bio-anthropology and Paleopathology, Institute of Forensic Medicine. Following the national laws for cultural heritages in Korea, the archaeological excavation was permitted by Hadong County and the related investigation was done by archaeologists in Jinju National Museum. The information on the permission and archaeological investigation was summarized by Hadong County as the county's official document (Munhwagwangwanggwa-27993, Oct 26, 2006).

Dating of the burial was based on ^14^C dating method, using Accelerator Mass Spectrometry (AMS) method. The analysis was carried out at the D-REAMS Radiocarbon Dating Laboratory, Weizmann Institute of Science, Israel.

The sample selected, RTT 5588, for ^14^C dating was mummified tissue which was pre-treated as organic material following the method in Yizhaq et al. [21]. Calibrated dates ranges were determined using the calibration program OxCal 4.1.7 (2010) [22], [23] based on Reimer et al [24] calibration curve.

Additional dating information was obtained from, the style of the clothing and the structure of the tomb.

The sex of the mummy was determined by the morphological structure of the pelvis (as seen on plane radiographs and 3-D CT images), mainly the open sciatic notch and the subpubic angle. Additional information on the sex of the mummy was gathered from her clothes (as males were usually dressed with pants and female with skirts). Age determination was based mainly on the stage of cranial suture closure (mainly the spheno-occipital), stages of epiphyseal closure in the long bones and ossification stage of the sacral vertebrae [25], [26]. Additional information on the age of the individual was gained via inspections of radiographic changes in the proximal femur [27].

### Macro- and microscopic analysis

#### Morphometric characteristics

A battery of 44 measurements was taken on the head, limbs and upper body segment, using standard anthropometric measurements and measuring devices (Table 1).

**Table 1:** Anthropometric data.

Measurements	From	To	Value (cm)
Anterior Trunk Height	Suprasternale	Pubic Symphysis	56.0
Thoracic Height	Suprasternale	Xiphoid	24.0
Abdominal Height	Xiphoid	Pubic Symphysis	31.3
Biacromial Breadth	Rt. Acromiale	Lt. Acromiale	30.6
Bicristal Breadth	Rt. Iliocristale	Lt. Iliocristale	29.5
Hand Length (R)	Stylion	Dactylion	16.6
Hand Breadth (R)	2nd Metacarpal	5th Metacarpal	6.6
Foot Length (R)	Longest Toe End	Foot Back End	19.9
Foot Breadth (R)	1st Metatarsal	5th Metatarsal	7.7
Width of the Head	Euryon	Euryon	14.9
Bitragion Diameter	Rt. Tragion (T)	Lt. Tragion (T)	14.4
Forehead Height	Vertex	Nasion	14.0
Special Height of the Head	Vertex	Endocanthion	9.9
Height of the Head and Nose	Vertex	Subnasale (SN)	17.4
Height of the Head and Face	Vertex	Gnathion (GN)	18.8
Length of Head	Glabella (G)	Opisthocranion (OP)	17.2
Circumference of the Head	Glabella (G)	Opisthocranion (OP)	54.1
Distance Vertex to Tragion (R)	Vertex	Tragion	15.2
Width of the Face	Rt. Zygion	Lt. Zygion	12.6
Width of the Mandible	Rt. Gonion	Lt. Gonion	11.9
Height of the Face	Nasion	Gnathion	10.5
Height of the Upper Face	Nasion	Subnasale	3.8
Height of the Lower Face	Subnasale	Gnathion	6.7
Lower Half of the Craniofacial Height	Euryon	Gnathion	13.7
Supraorbital Arc	T-G-T	T-G-T	23.6
Maxillary Arc	T-SN-T	T-SN-T	21.6
Mandibular Arc	T-GN-T	T-GN-T	23.5
Height of the Mandibular Ramus (R)	Gonion	Condylion laterale	6.4
Height of the Mandibular Ramus (L)	Gonion	Condylion laterale	6.6
Intercanthal Width	Rt. Endocanthion	Lt. Endocanthion	3.2
Biocular Width	Rt. Exocanthion	Lt. Exocanthion	9.1
Length of the Eye Fissure (R)	Exocanthion	Endocanthion	3.1
Length of the Eye Fissure (L)	Exocanthion	Endocanthion	3.3
Orbito-Aural Distace (R)	Exocanthion	Otobasion Superius	7.1
Orbito-Aural Distace (L)	Exocanthion	Otobasion Superius	6.7
Orbito-Tragion Distace (R)	Exocanthion	T	7.5
Orbito-Tragion Distace (L)	Exocanthion	T	7.7
Orbito-Gonial Distace (R)	Exocanthion	Gonion	11
Orbito-Gonial Distace (L)	Exocanthion	Gonion	11.4
Orbito-Glabellar Distace (R)	Exocanthion	Glabella	5.8
Orbito-Glabellar Distace (L)	Exocanthion	Glabella	5.9
Height of the Orbit (R)	Otobasion Superius	Otobasion Inferius	3.8
Height of the Orbit (L)	Otobasion Superius	Otobasion Inferius	3.6
Anatomical Width of the Nose	Rt. Alar point	Lt. Alar point	3.0

#### Radiological examinations

**Figure 5:**
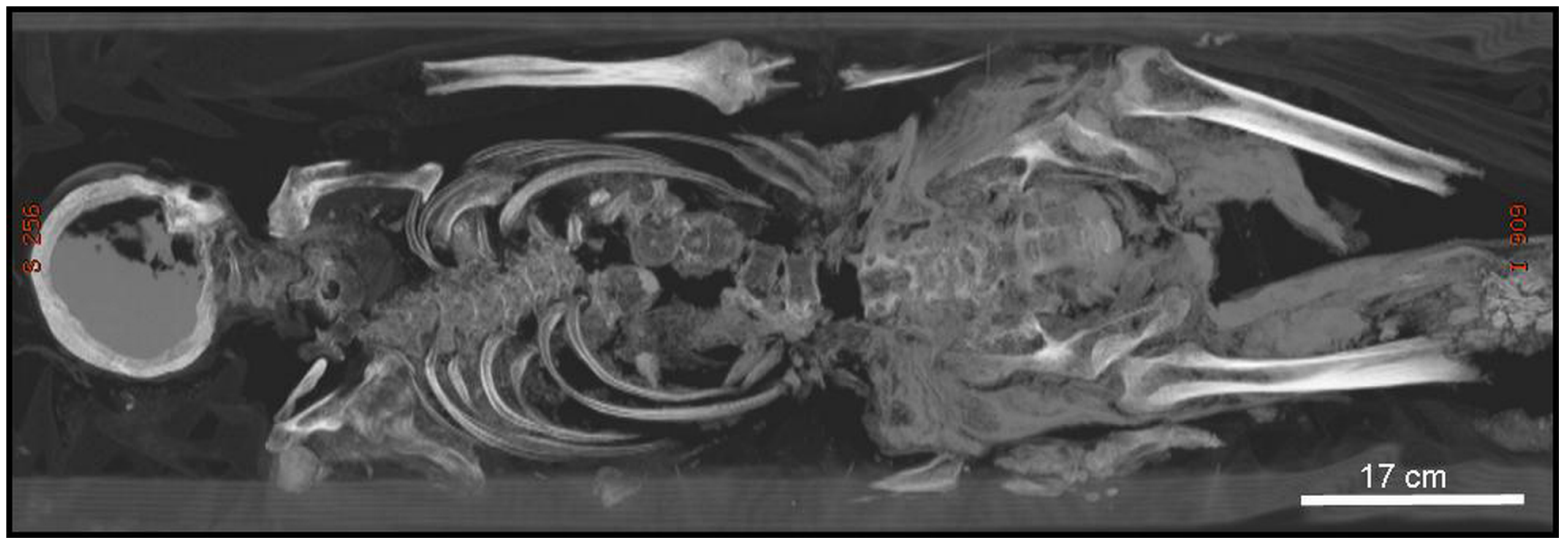
Full-body computed tomography image of the Hadong I mummy.

A CT scan of the mummy was performed at the Department of Diagnostic Radiology at Dankook University Hospital, using a 64-slice Multidetector Computed Tomography (MDCT) scanner (VCT; General Electric Medical Systems, Milwaukee). A spiral volume was acquired from the head to above the knee joint (1811 images), and from the head to the mandible (160 images). The scanning parameters used were: slice thickness  = 0.625, and KVp  = 120. The axial source images were transferred to two independent workstations (Advantage Windows Workstation 4.3; General Electric Medical System, Milwaukee, USA, and Philip Brilliance 64, Philips Medical Systems, Cleveland, OH, USA) for post-processing by two independent research teams (Korean and Israeli). For detailed analysis, multi-planar reformatting (MPR) and volume rendering (VR) techniques were applied. The cranial, thoracic and abdominal segments, as well as most of the extremities of the mummy, were relatively well preserved. Some loose parts, especially the right upper limb and both distal parts of the lower limbs (below the distal femoral diaphyses) were not scanned with the main body (Fig. 5).

#### Dissection of the mummy

**Figure 6:**
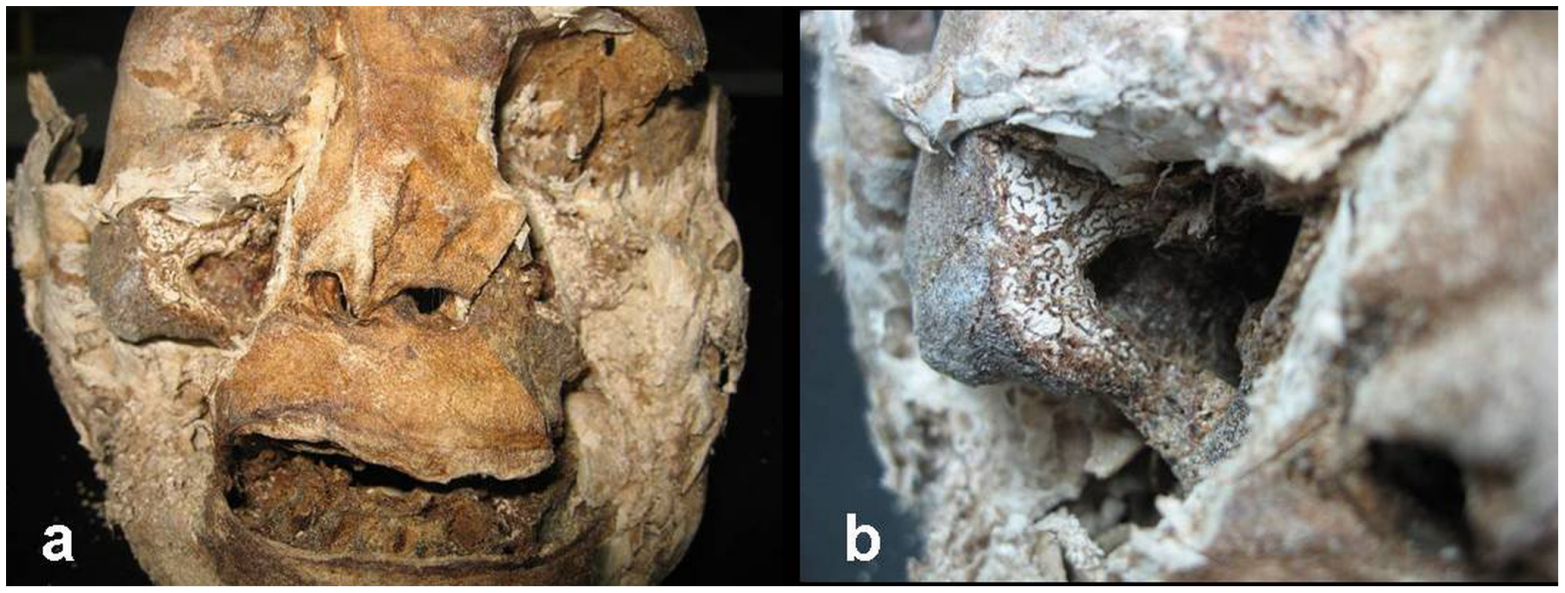
The head of the Hadong I mummy. (a) Overview of the head demonstrates prominent diffuse expansion of the lower portion of the face. (b) Section through the maxillary sinus. Note the hypertrophic zygomaxillary area (which results in the classic swollen-cheeked phenotype in living patients), thickened orbital floor (which results in upturned eyes in the living), cortical thinning, whorled pattern of the woven bone, and deformity of the maxillary sinus.

Following CT scanning, the mummy was moved to the Anthropology Laboratory, Seoul National University College of Medicine (SNUCM), for further anatomical and anthropological inspections. Initially, a thorough visual physical examination of the body was performed and the entire body was examined with a magnifying self-lightening glass. The mummy was then dissected starting at the head and down into the pelvic region following standard anatomical/pathological procedures. During dissection, all identified structures (organs) were compared with the CT findings. Unrecognized organs were sampled for histological analysis for further identification. Due to excellent soft tissue preservation, direct access to the actual bones was limited. As a result, CT scans were relied upon for many of the gross observations on the bones. Finally, a large segment of the right zygomaxillary area was exposed and sectioned for gross observation of tissue arrangement and sinus wall morphology, as well as histological examination (Fig. 6).

#### Histological examinations

Three samples of bone tissue were removed from the anterior wall of the maxillary sinus for microscopic examination. One section was prepared for light microscopy and stained with hematoxylin and eosin (H&E) [28]. Another section was prepared for transmission electron microscopic study. The third section was utilized for surface analysis, chemical analysis, and imaging using an Environmental Scanning Electron Microscope (ESEM), with energy dispersive spectroscopy (EDS) (Quanta 200, FEI Company).

### Ancient DNA analysis

The DNA analysis was carried out parallel to the morphological analysis. The ancient DNA (aDNA) studies were carried out in dedicated aDNA laboratories both in Israel and Korea. Twelve samples for ancient DNA (aDNA) analysis were taken from the mummified brain within the intact calvarium, following division of the brain tissue into 15 equal portions (∼10 mg each). DNA was extracted from all samples using two different methods: guanidinium thiocyanate (GuSCN) followed by silica capturing [29], [30] and Phenol – Chloroform [31]. To minimize the presence of fat in the extracts, the tissue samples were treated prior to the extraction, and the fats physically removed under the microscope. Six DNA extraction attempts were made with two different samples of the brain, resulting in a total of 12 DNA samples being analyzed.

As a tentative diagnosis of cherubism was made based on morphological criteria, we attempted to amplify the mutation associated with the disease along two regions of the SH3BP2 and PTPN11 genes, using published primer sets [13], [32]. In addition, we amplified the hypervariable region I (HVR-I) of the human mitochondrial control to determine the origin of the mummy and indicate the presence/absence of DNA in the extract [33]. The primers were designed to amplify regions that do not exceed 300 bp. PCR was performed in a total volume of 25 µl which included 10× PCR buffer, 0.25 mM of dNTPs, 2.5 mM of MgCl_2_, 0.4 µM of each primer and 0.25 U/reaction of AmpliTaq Gold [Applied Biosystems, Inc], using a touchdown PCR method. The PCR consisted of an initial denaturation at 95°C for 10 min, followed by a total of 45 cycles of 15 sec denaturation at 94°C, 45 sec annealing for two cycles each at 60°C, 58°C, 56°C, 54°C, 52°C, and 35 cycles at 50°C or 48°C, 45 sec elongation at 72°C, and a final elongation step of 10 min at 72°C. PCR products were eluted using electrophoresis to detect successful amplifications. To optimize the amplification success rate, the amplification was carried out under various conditions, such as differential MgCl_2_ and DNA concentrations.

In order to avoid contamination with contemporary human and/or animal DNA, all pre-PCR experimental steps (e.g., sampling, DNA extraction and PCR set-up) were carried out in a dedicated aDNA laboratory, located in the Faculty of Agriculture at The Hebrew University of Jerusalem, Israel. Each pre-PCR step was performed in a different hood equipped with sterile tubes, reagents/solutions, and pipettes, as well as disposable filtered tips in order to prevent cross-contamination between samples. In addition, the PCR mixture, excluding the *Taq* polymerase, was UV cross-linked to destroy any possible contaminants. Multiple blank extraction and blank PCR controls were included to insure the absence of contamination during the extraction and amplification procedures. The PCR amplification and post-PCR analyses were later conducted in the modern DNA laboratory, located in a building physically separate from the aDNA facility.

## Results

### Preservation

The head and trunk of the mummy were relatively well preserved, whereas the preservation of the extremities was relatively poor, except for the hands and feet. A mummified brain remained within the cranial cavity as did mummified lung tissues in the thorax and part of the intestines in the abdomen [18]. The taphonomical process within this unique environment (encapsulated double coffin) has recently been described in a separate paper [34].

### Date of burial

The radiocarbon age obtained is 280±35 year uncal BP. The calibrated time ranges for 68.2% probability (±1 standard deviation) were 1520AD (39.3%) 1575AD and 1630AD (28.9%) 1660AD. For 95.4% probability (±2 standard deviation) we obtained the following ranges: 1490AD (56.5%) 1603AD, 1615AD (35.4%) 1670AD and 1785AD (3.5%) 1800AD.

From probability distribution of the calibrated ranges, the most probable range for RTT 5588 is the 16^th^ century AD to the first half of the 17^th^ century AD.

The evidence from the style of clothing in which this individual was dressed conformed to the fashions of the early 17^th^ century.

### Sex, age and physical appearance

**Figure 7:**
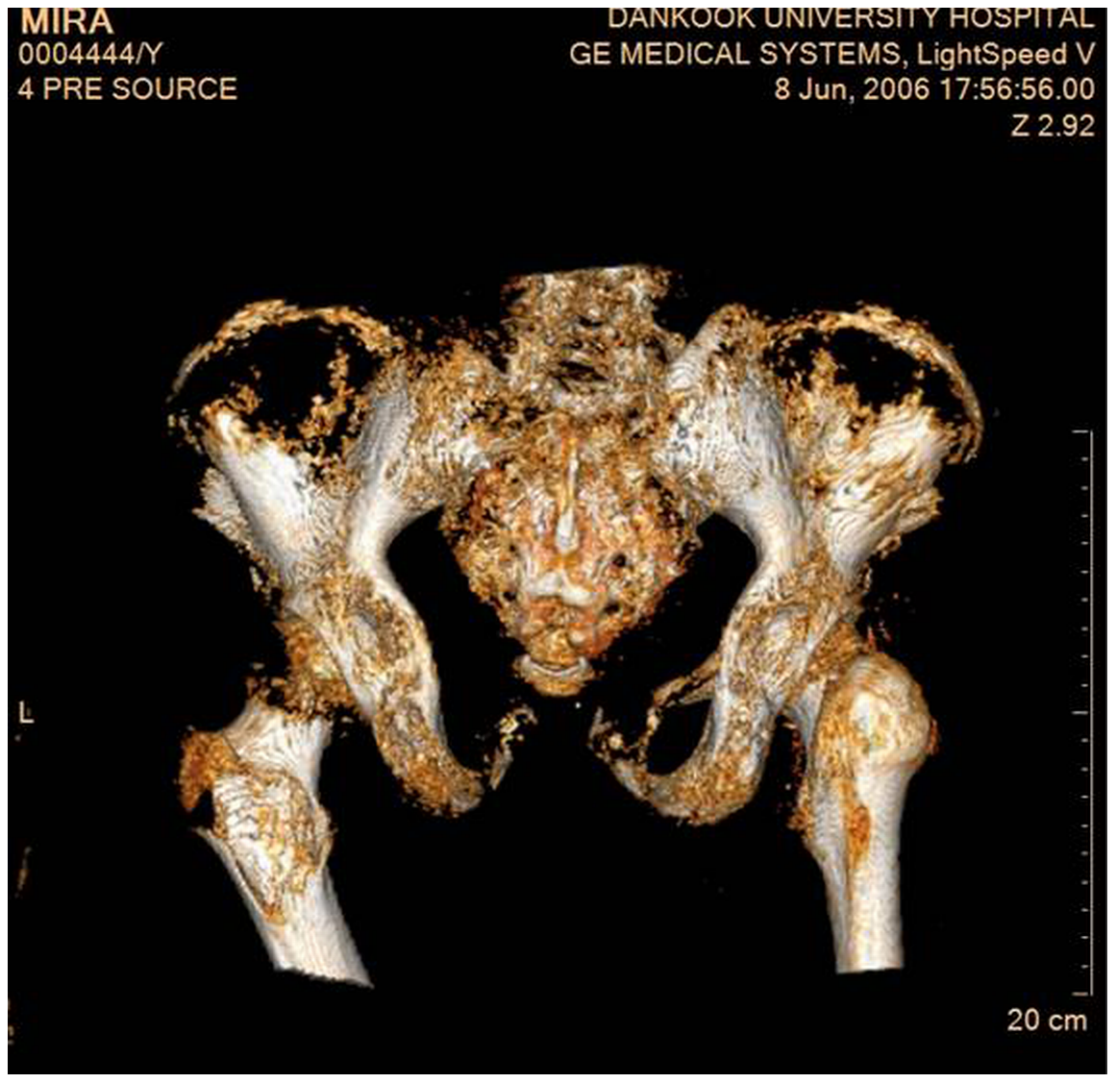
Morphological characteristics of the pelvis of Hadong I mummy indicating a female sex (posterior view): note the wide-open sciatic notch and the angles of the inferior pubic rami.

Based on several key morphological characteristics (Fig. 7), this was most probably a young female: This was further confirmed by the style of her clothes. The approximate age of the individual at the time of death was between 18 and 25 years. Detailed anthropometric observations are presented in Table 1.

### Distribution of pathology

Only the maxilla and mandible exhibited pathological osseous changes. All the long bones appeared normal, without evidence of periosteal reaction or intra-osseous lesion. Calvarial bones were also normal in shape and thickness.

### Characterization of pathology

**Figure 8:**
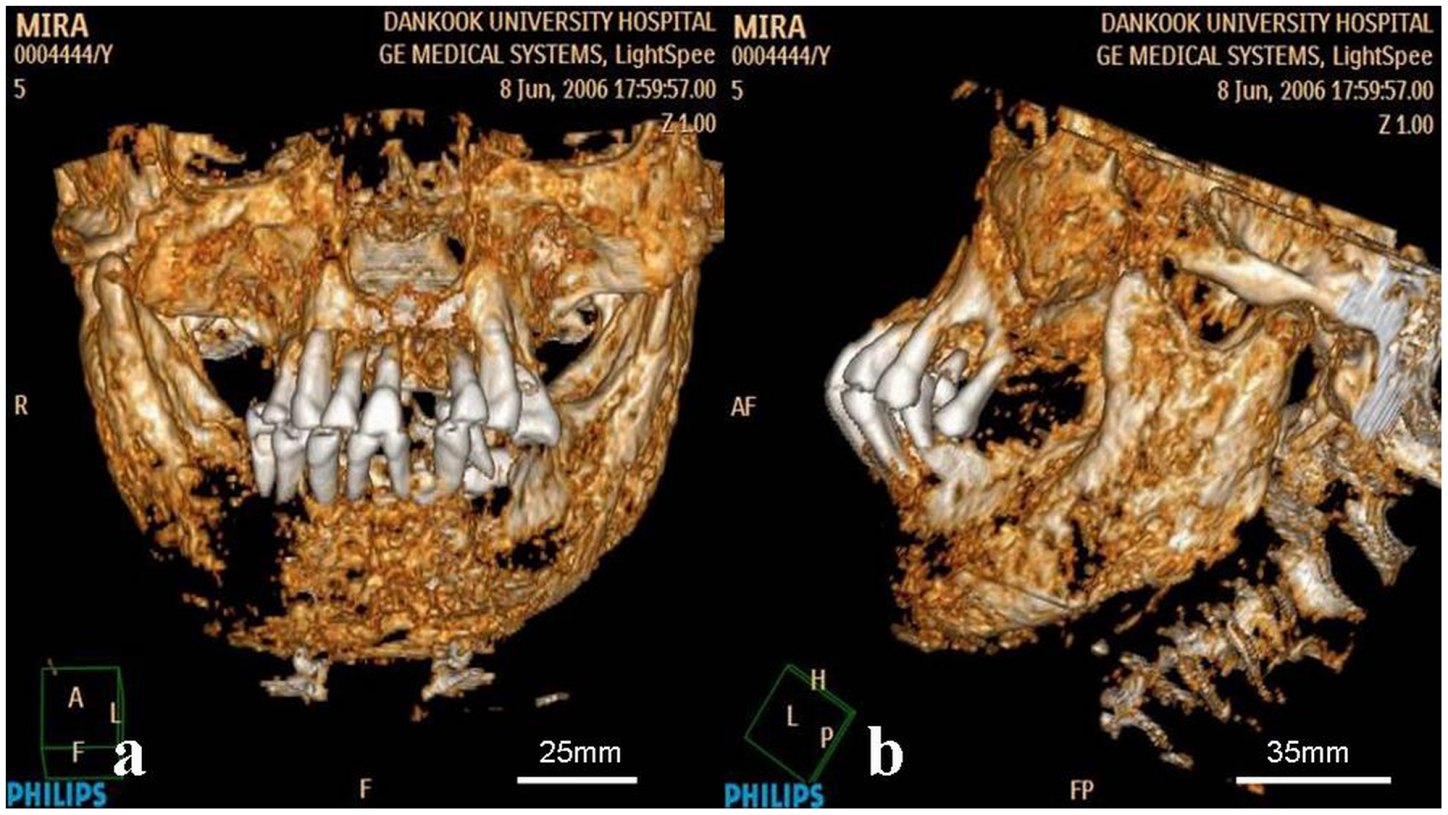
3-D volume rendering of the mummified head. Frontal (a) and lateral (b) projections demonstrate diffuse symmetrical expansion of the mandible and maxilla. Note the broadened mandibular body and ascending ramus with shallow mandibular notch, the expanded coronoid process with blunt margins, absence of posterior teeth, displaced permanent teeth secondary to the jaw lesions, abnormal crown-shaped teeth, dental root resorption and malocclusion.

**Figure 9:**
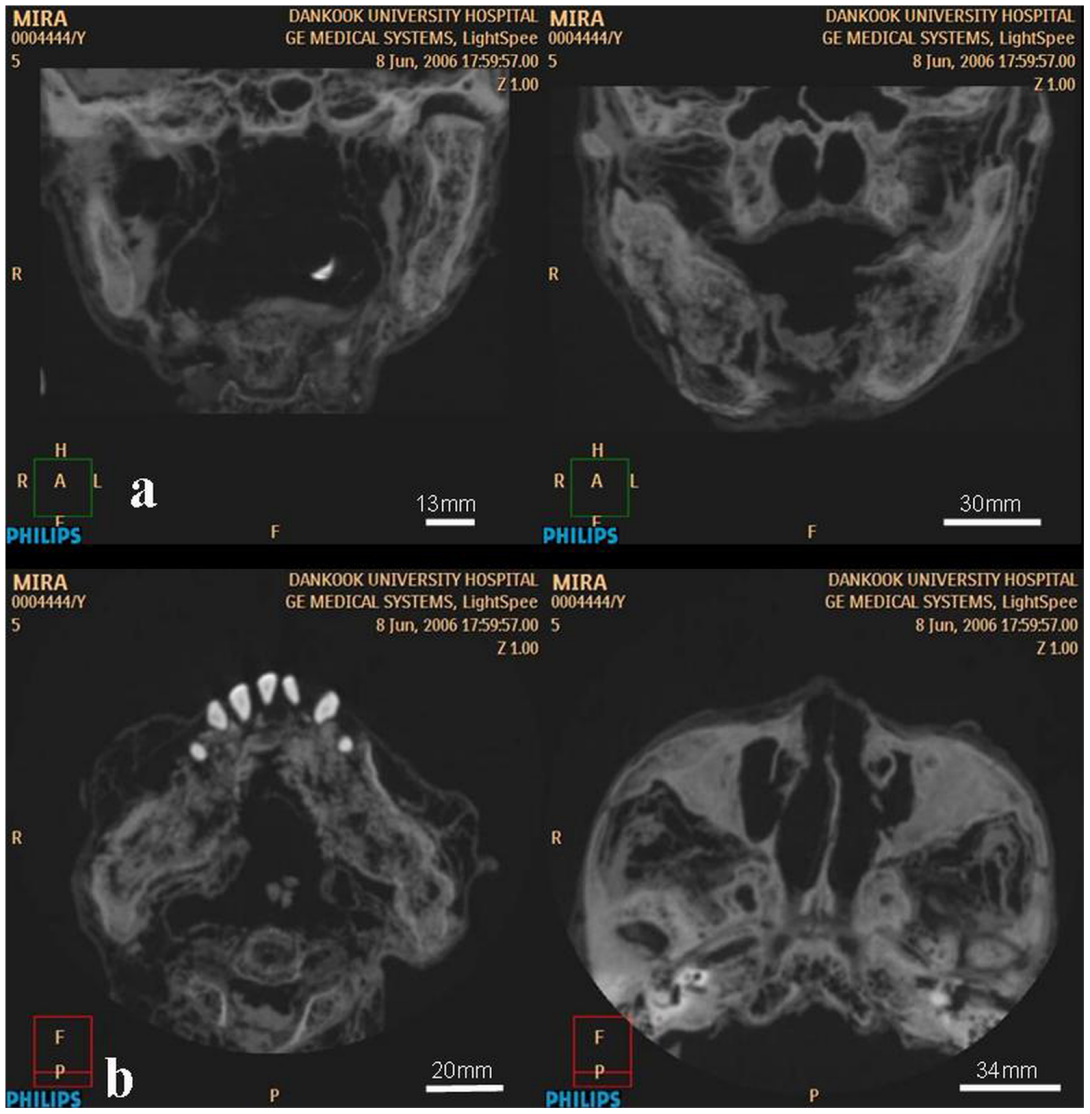
CT slices through the level of the maxilla and mandible. (a) Coronal images demonstrate symmetric, “bubbly” expansion of the maxilla (including orbital floor and maxillary buttresses) and mandible (with diffuse involvement of the body, ascending ramus and condyle). (b) Axial images demonstrate marked expansion of the mandibular body and ramus, disorganized spacing of the teeth (with missing molars), and thickening of the zygomaxillary area.

**Figure 10:**
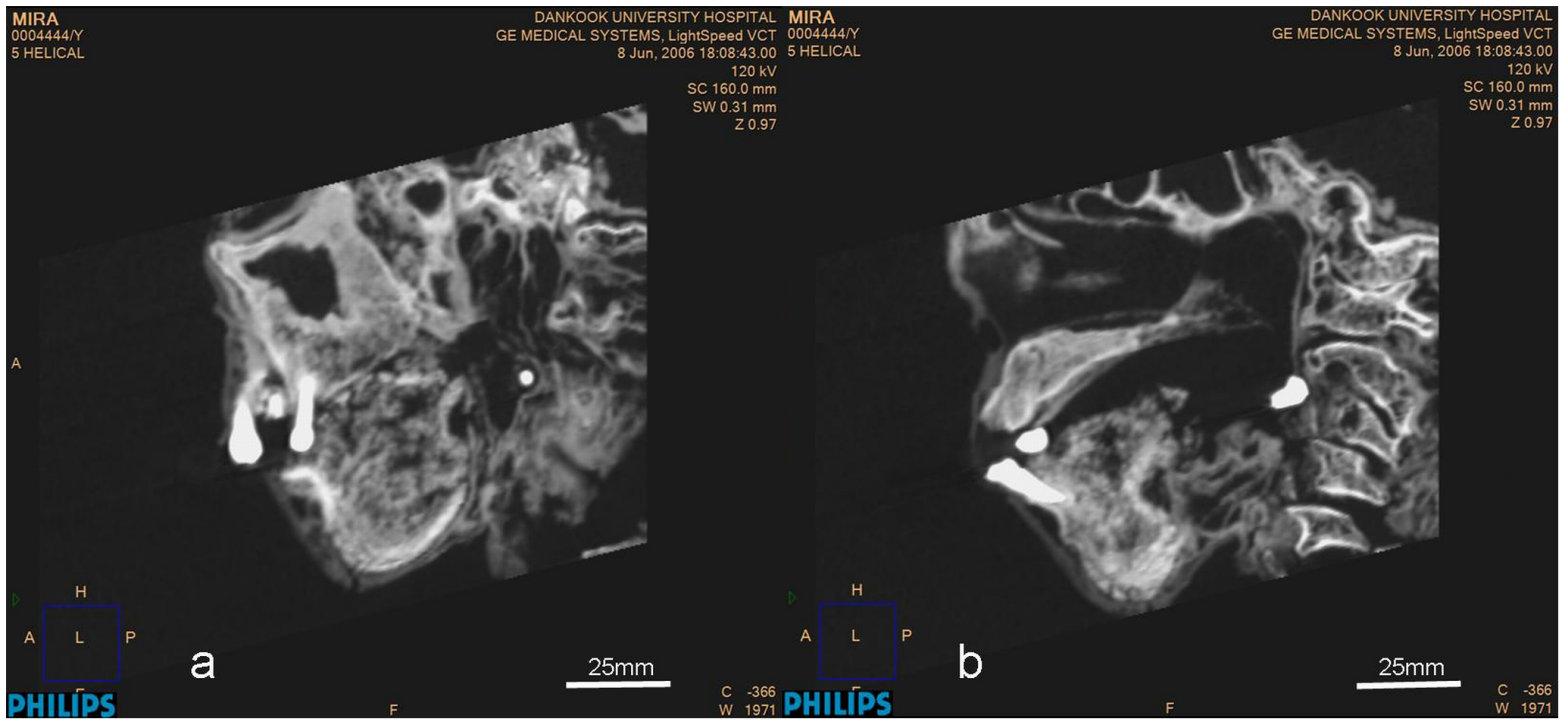
Sagittal (a) and parasagittal (b) CT sections through the lower face. The images demonstrate thickening of the maxillary walls and orbital roof, near-disappearance of cortical bone in the mandible, and resorption of dental roots.

**Figure 11:**
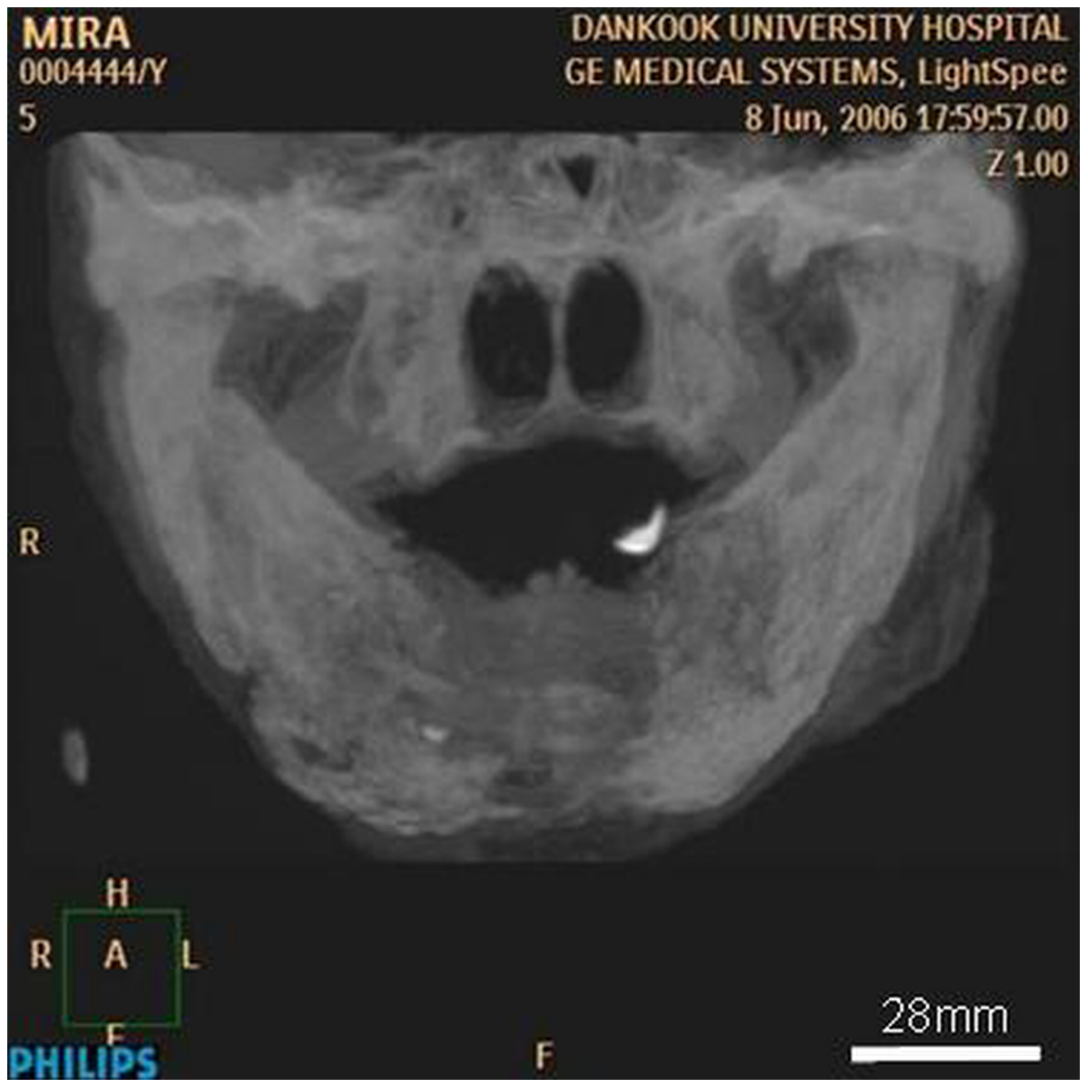
Radiographic image of the jaws demonstrates heterogeneous sclerosis of the bone, corresponding to the classic “ground-glass” appearance. Note the radiolucent shadow at the mental area.

The macroscopic osseous changes are characterized by bilaterally symmetric expansile remodeling of the mandible and maxilla, including the ascending rami and coronoid processes (Figs. 8–11). Although the left condyle is slightly flat, the overall morphology of the condyles is normal. In profile, the lower face looks concave, due mainly to extensive projection of the subnasal region anteriorly (Fig. 8B). CT imaging confirms the presence of expansile remodeling of the involved bones, with hypertrophy of the maxillary sinus walls, thinning of the cortices, coarse trabeculation and multilocular intraosseous radiolucencies (Figs. 9B,10). Radiographs also show typical patchy ground glass haziness, representing heterogeneous sclerosis interspersed with lytic lesions (Fig. 11). The maxillary sinuses are deformed, with associated mural thickening (Fig. 10). Coronal images show slight bulging of the orbital floors by the over-thickening of the roofs of the maxillary sinuses. Dental abnormalities include absence of all molars, tooth displacement, and irregularly spaced dentition,(Figs. 8,9B,10). Some of the teeth demonstrate abnormal shape and structure: the upper left first premolar and the lower left lateral incisor appear small and atypical (Fig. 8). Malocclusion is marked, presenting as severe proclination of both upper and lower teeth, with an edge-to-edge relationship of the incisors. The left lower first premolar is in a lingual ectopic position creating scissors bite with the upper left first premolar. A cervical lesion is present in the lower right first premolar (in the distal aspect) and lower right second premolar (mesial aspect). Occlusal attrition can be seen especially in the incisors region. The upper left central incisor has fallen from its socket postmortally and appears in the CT in a different location.

**Figure 12:**
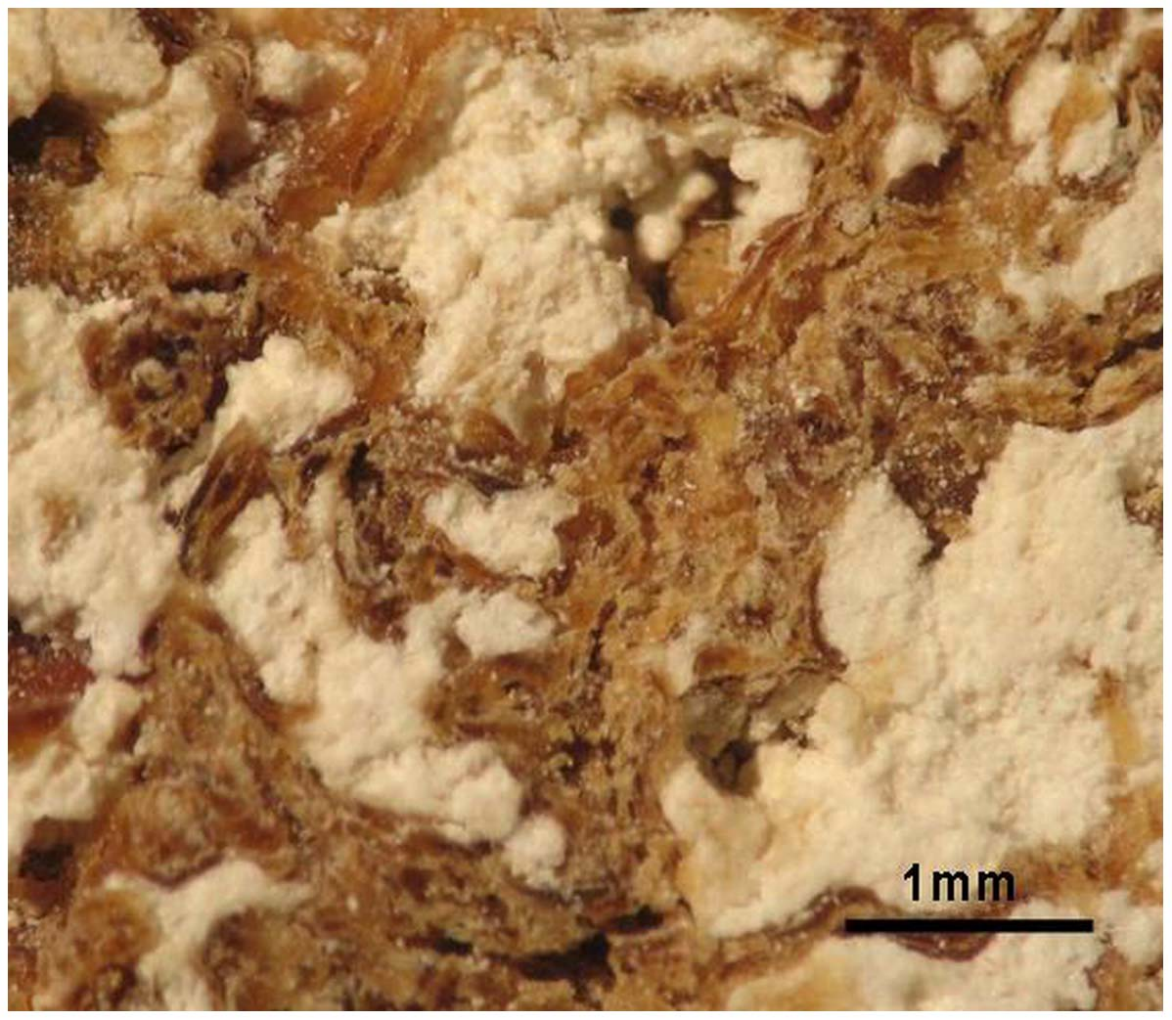
Low magnification of the sectioned area demonstrates amorphous islands of white material occupying the spaces between the brownish material.

**Figure 13:**
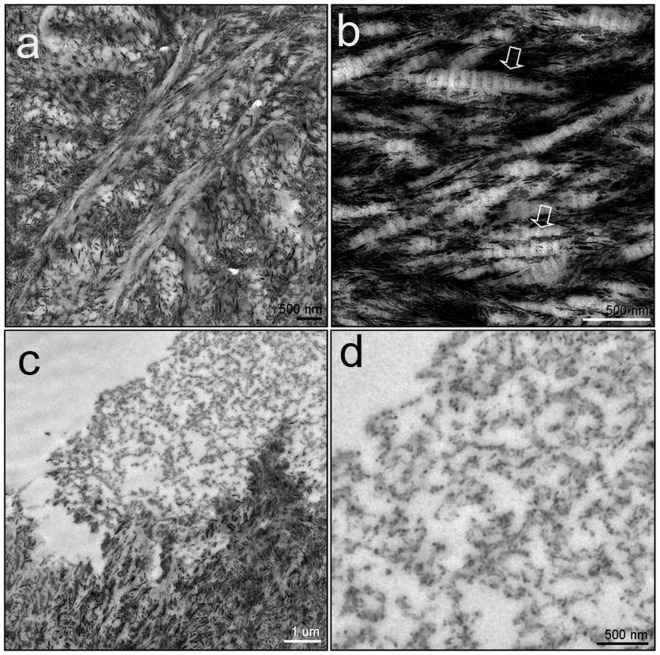
Electron microscope images of the sectioned area shown in Fig. 12. The brownish material seen in figure 12 represents bony trabeculae. Hydroxyapatite crystals are oriented parallel to the long axis of irregularly arranged collagen fibers (A and arrows in B). The whitish material seen in Fig. 12 is amorphous in nature and composed of both organic and inorganic material (C and D).

**Figure 14:**
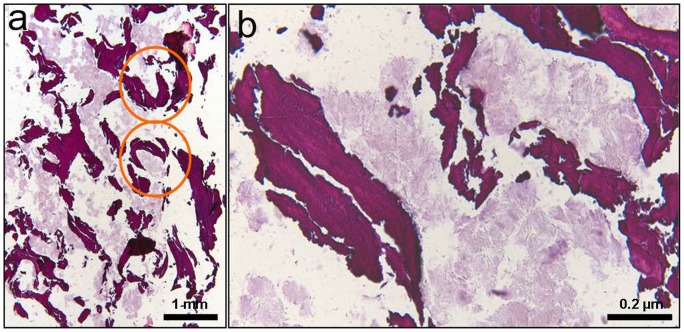
H&E stained histological sections of the maxillary sinus wall. (B) is a magnified image of (A). Intensely stained (trabecular bone) and weakly stained areas (amorphous material) are distinctly seen. Note the “C-shaped” trabeculae of the woven bone (orange circles) dispersed within the lightly stained stroma.

**Figure 15:**
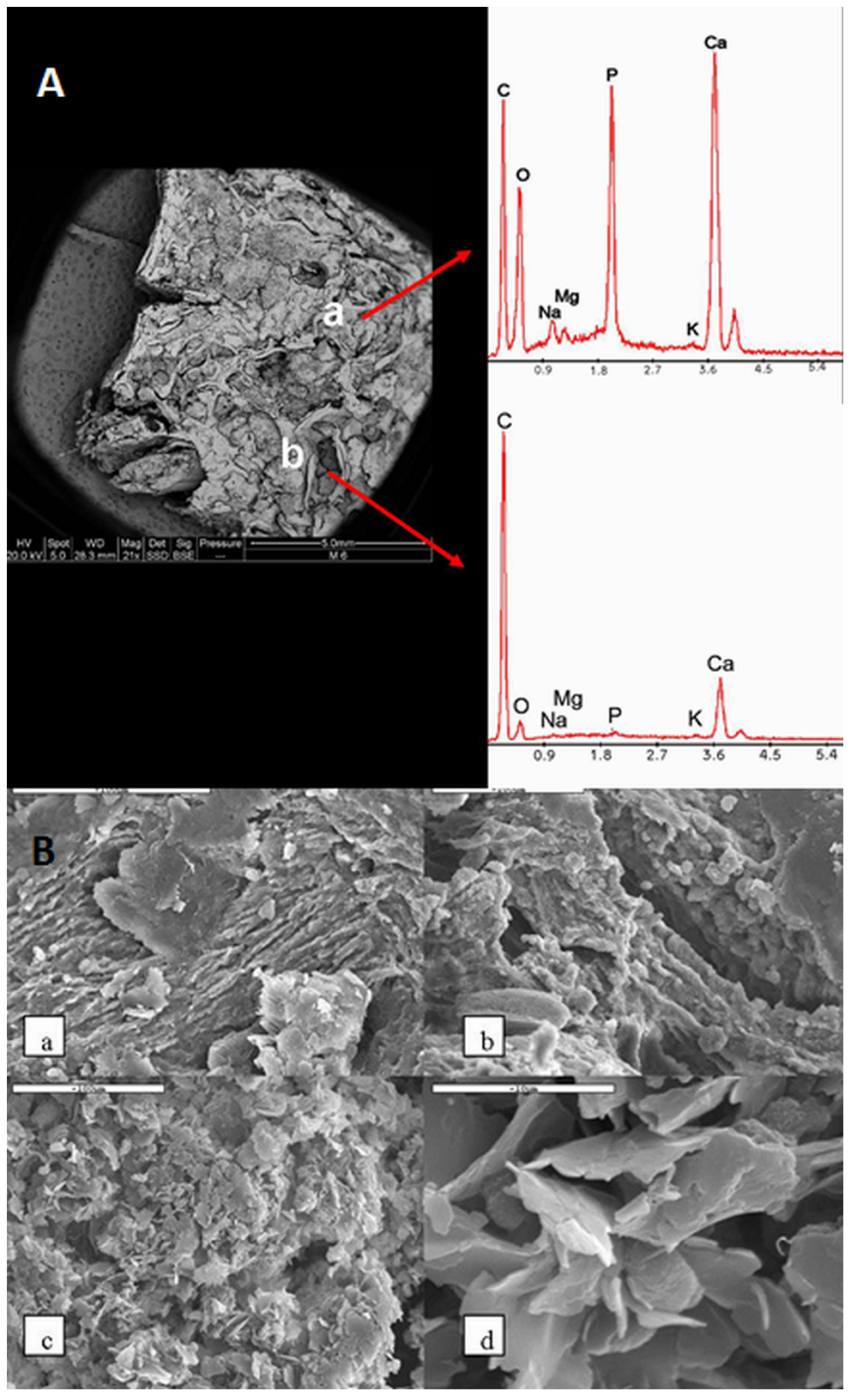
Surface and chemical analysis of the maxillary sinus wall. **A.** Surface and chemical analyses of bone section from the zygomaxillary area using ESEM. Backscattered electron image (left) - bright phases on the backscattered image composed, based on X-ray EDS spectra, of bony material (a =  bone trabeculae), dull phases (b =  cysts filled with partially calcified fibrous tissue), mainly of organic material. Noteworthy is the relatively high concentration of organic material within the bony trabeculae (right top) and the presence of calcite crystals coating some of the organic material (bottom right). “C” and “Y”-shaped trabeculae are clearly visible. **B.** SEM clearly showing that the “brownish” parts (a and b) are composed of bony trabeculae, whereas the “whitish” part (c and d) contains crystals admixed with organic material.

**Figure 16:**
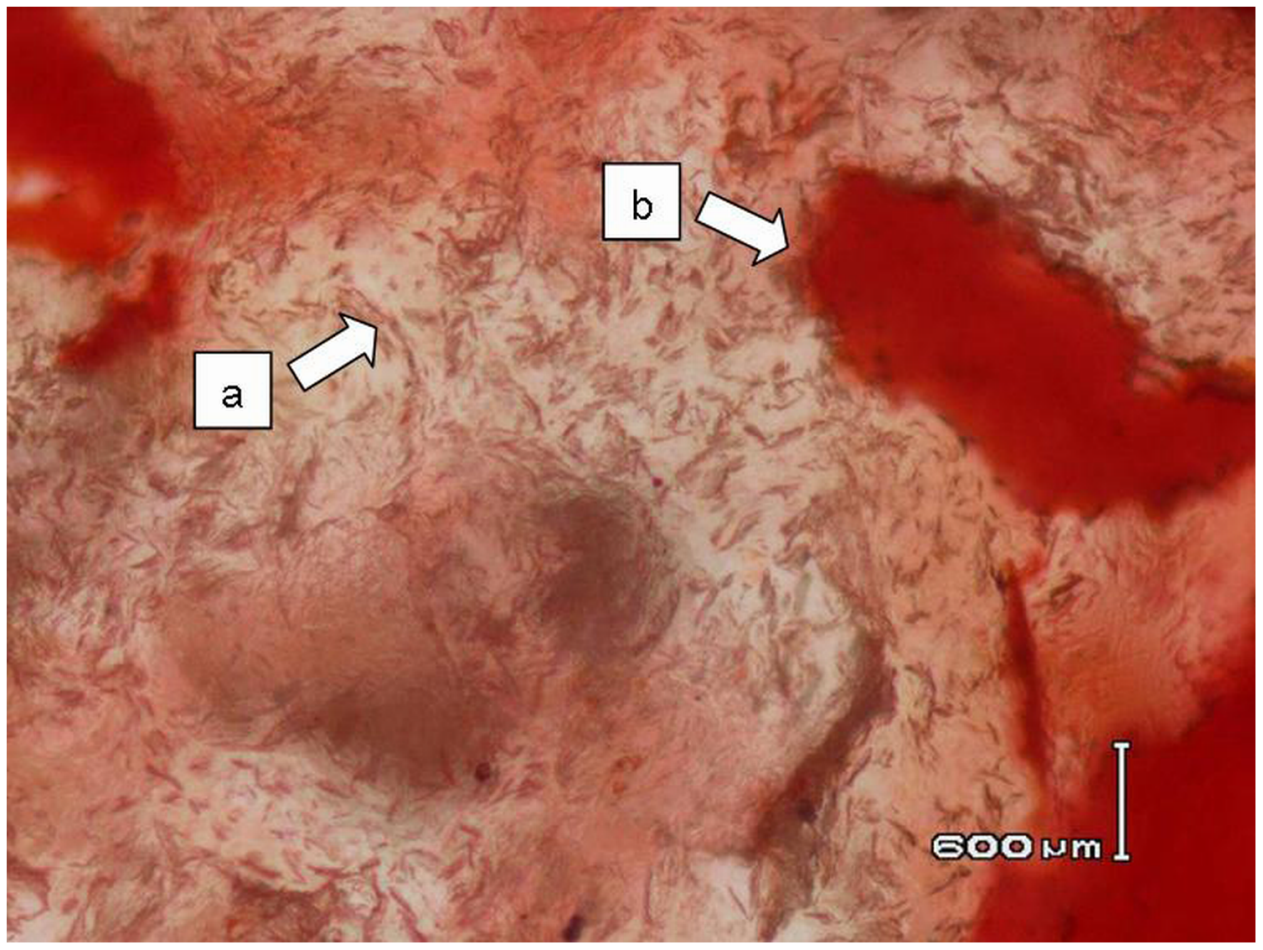
Histological section (H&E staining) shows cysts filled with fibrous tissue and crystals (a) and surrounded by bony spiculae (b).

**Figure 17:**
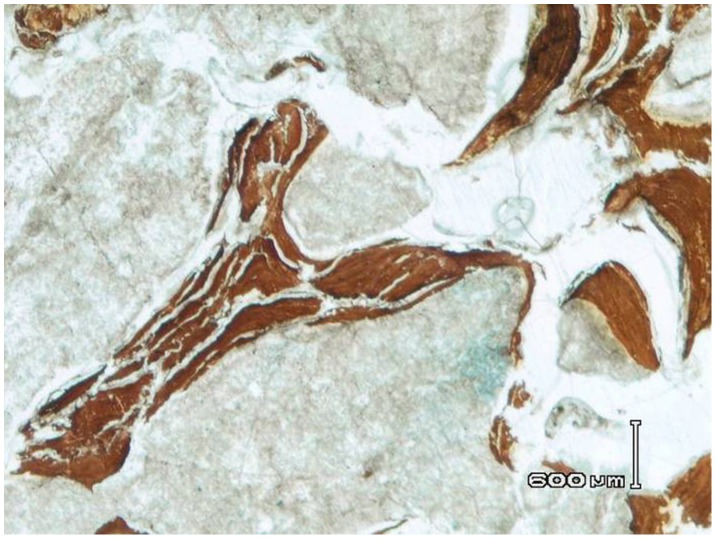
“Y” shaped bony trabeculae.

Microscopic evaluation of the sectioned area under low magnification revealed two distinct components within the bony matrix: a brownish material, organized in a beehive-like pattern, and a white amorphous material which fills the spaces between the brownish stripes (Fig. 12). Histological (Figs. 13,14) and chemical analysis (Fig. 15A,B) show that the brownish material corresponds to bony trabeculae and the white amorphous material represents a mixture of organic and inorganic material, most likely cysts filled with partially calcified fibrous tissue (Figs. 14,15). “C” and “Y”-shaped trabeculae are clearly visible (Figs. 14–17). The microscopic view as seen in histological sections shows varying degrees of immature to mature collagen whorls surrounded by circular fragments of immature (woven) bone.

### aDNA analysis

In order to definitively diagnose cherubism in our subject, attempts (in two separate laboratories, in Korea and in Israel) were made to amplify the corresponding mutations in the *SH3BP2* gene in all 12 DNA samples, regardless of the extraction method used. Efforts were made to reduce the fatty component within the extracts in order to prevent it from inhibiting the amplification process. Whenever DNA amplification was unsuccessful, an attempt was made to optimize the PCR process by varying the concentration of Mg and/or DNA in the sample. The presence of primer dimer in all the PCR products, as well as positive amplification of the control, suggests the absence of DNA in the samples. Failure to amplify the hypervariable region HVR-1 further supports the absence of viable DNA extracts.

## Discussion

### Diagnosis

The diagnosis of cherubism in this 17^th^ century mummy is based on the presence of multiple histological and radiological criteria typical of the disease. Macroscopically, these findings primarily include pathologic involvement anatomically restricted to the upper and lower jaw (sparing the condyles), with bilaterally symmetric expansile remodeling of the maxilla and mandible, mural hypertrophy of the maxillary sinus, cortical thinning, coarse trabeculation, and sclerotic “ground-glass” haziness interspersed with lytic lesions on CT imaging [2]–[9]. Dentally, the syndrome affects the developing permanent teeth, causing displacement, non-eruption or absence of teeth, together with malocclusions [4], [7], [35], all of which can be noticed in this case. The upper left first premolar and the lower left lateral incisor appear to be small and atypical, which is pathognomonic for the syndrome. All molars are missing, which coincides with the progress of the disease, since the area of cysts interferes with the formation of first and second molars [36], and might cause root resorption of the teeth in the area of the cyst, consequently leading to tooth loss (e.g.,first molar, upper right second premolar and lower left second premolar) [37].

Microscopically, the histology shows a matrix of delicate bony trabeculae surrounded by an amorphous material. This pattern is consistent with cherubism: the ‘C-shaped’ trabeculae of woven bone dispersed within a fibrous stroma, including abundant collagen fibers of variable maturity [38]–[41].

### Differential diagnosis

Primary differential considerations in this case include the entire series of benign bony lesions involving the mandible. These include craniofacial fibrous dysplasia, Jaffe-Campanacci syndrome (multiple non-ossifying fibromas), brown tumors of hyperparathyroidism, central giant-cell granuloma, and familial gigantiform cementoma [8], [10].

The main differential diagnosis of cherubism is the craniofacial form of fibrous dysplasia, another benign fibro-osseous condition. Fibrous dysplasia involves defective differentiation of osteoblasts, with subsequent replacement of the normal marrow and cancellous bone by immature bone and fibrous stromae [42]. Given the radiologic similarities between these two entities, namely “bubbly” multiloculated ground-glass bony lesions, cherubism has long been erroneously dubbed fibrous dysplasia of the jaw. Numerous clinical and radiographic parameters distinguish between these two processes, and genetic analysis has proven them to be distinct on the molecular level as well. While both diseases predominate in childhood, fibrous dysplasia usually presents later (10–30 y.o.) and continues to progress past adolescence, while cherubism typically presents before 5 years of age and begins to regress spontaneously at puberty [9]. In general, the lesions of fibrous dysplasia are more asymmetric, tend to be monostotic, and may involve any bony structure. In the specific craniofacial form of the disease, those lesions which are polyostotic tend to be unilateral, and the maxilla is favored over the mandible [43], [44]. In contrast, cherubism is typified by symmetric bilateral disease of the lower jaw with or without maxillary involvement. Furthermore, patients with fibrous dysplasia usually do not present with the swollen cheeks, upward turning of the eyes, or dental derangement that typify the cherubism phenotype [3], [8], [10], [42], [43]. Histologic samples of fibrous dysplasia yield irregular spindles of woven bone, usually nonmineralized and dispersed within a fibrocellular matrix. The multinucleated giant cells which predominate in cherubism are rarely seen in fibrous dysplasia [7], [41].

Central giant-cell granuloma, formerly called giant cell reparative granuloma, is a rare, benign intraosseous disorder that usually occurs in the mandible of children and young adults, more commonly females. This can also lead to facial deformity and dental displacement, and was renamed when it was found to be an essentially destructive rather than reparative process. Histologically, the lesions consist of cellular fibrous tissue containing multiple foci of hemorrhage, clusters of multinucleated giant cells, and occasionally trabeculae of woven bone [45]. Despite the histologic similarities, cherubism can be distinguished from this disease radiologically, because the majority of lesions in central giant-cell granuloma are unilocular, whereas cherubism is usually a multilocular process [46]. It should be noted that cherubism and central giant-cell granuloma are considered histologically indistinguishable, and “should no longer be linked with fibrous dysplasia.” [3]

Brown tumors are benign lytic giant-cell lesions that arise rarely in the bone tissue as a consequence of hyperparathyroidism. These can theoretically occur in both the maxilla and mandible, but are relatively rare in the jaw overall. The age of onset is usually in adulthood, versus childhood in cherubism. Brown tumors are an unlikely diagnosis in the absence of biochemical or ancillary radiologic signs of hyperparathyroidism [9], [47].

While the very rare Jaffee-Campanacci syndrome also involves expansile polyostotic lytic lesions (non-ossifying fibromas), this can be excluded from diagnostic consideration, as the multi-loculated sub-cortical lesions characteristic of the disease usually spare the jaws (they appear mainly in the femur and tibia).

Familial gigantiform cementoma (FGC) is a rare disorder characterized by the production of cementum in its osseous lesions. In contrast to cherubism, these lesions predominate in the maxilla rather than the mandible, and is not symmetrical. Additionally, in FGC.enlargement of both mandibular and maxillary masses may cause severe disfigurement of the face and obstruction of the nasal cavity and maxillary sinus, rarely seen in cherubic persons [7], [9], [48], [49].

In pinpointing the diagnosis of this case it is noted that, of the pathologies delineated above, only cherubism shares the macroscopic hallmark features of our subject, namely: pathology limited to the jaw, bilaterally symmetric enlargement of the lower and upper jaws, and absence of periosteal reaction. Finally, it is noteworthy that several cases of cherubism have actually been reported in the modern Korean population [50], [51].

## Summary

While it was technically non-feasible to extract viable DNA from our subject for diagnostic amplification of the *SH3BP2* gene, the morphological, radiographic, and histological features of this 17^th^ century Korean mummy suggest that this represents the first documented case of cherubism in a historic population. Considering the age of this female and the sclerotic involution of the jaws as revealed radiographically, the disease was likely in its regresive phase.
